# Supplementary material for: Blood group typing from whole-genome sequencing data
Source: PLoS One. 2020 Nov 12;15(11):e0242168. doi: 10.1371/journal.pone.0242168 (PMC7660531; doi:10.1371/journal.pone.0242168)
Supplement: S2 Table — Mean [min-max] number of reads and estimated read depth for each blood group gene analyzed. For each locus, gene size and effective size (i.e. sequence without repeated patterns in intronic sequences) are given. (DOCX) [file pone.0242168.s002.docx]

**Supporting Table S2. Number of reads and read depth.** Mean [min-max] number of reads and estimated read depth for each blood group gene analyzed. For each locus, gene size and effective size (i.e. sequence without repeated patterns in intronic sequences) are given.

| Locus | Size | Effective size | Reads | Read depth |
| --- | --- | --- | --- | --- |
| *KEL* | 28303 | 19179 | 1445 [583-2721] | 11 [5-21] |
| *ACKR1* (FY) | 8781 | 8487 | 552 [224-1176] | 10 [4-21] |
| *SLC14A1* (JK) | 72496 | 38371 | 2637 [1028-5025] | 10 [4-20] |
| *ACHE* (YT) | 13140 | 10446 | 677 [316-1563] | 10 [5-22] |
| *ART4* (DO) | 21169 | 11820 | 880 [381-1304] | 11 [5-17] |
| *CD44* (IN) | 100533 | 67799 | 5313 [2337-9360] | 12 [5-21] |
| *AQP1* (CO) | 20664 | 17368 | 1269 [648-2690] | 11 [6-23] |
| *SLC4A1* (DI) | 26746 | 18238 | 1374 [678-3220] | 11 [6-26] |
| *ICAM4* (LW) | 8549 | 6508 | 480 [200-1243] | 11 [5-29] |
