# Supplementary material for: Blood group typing from whole-genome sequencing data
Source: PLoS One. 2020 Nov 12;15(11):e0242168. doi: 10.1371/journal.pone.0242168 (PMC7660531; doi:10.1371/journal.pone.0242168)
Supplement: S3 Table — Typing status according to number of reads (mean [min-max]) (No.: Number) and read depth (mean [min-max]); (Incorrectly typed samples could not be included in the statistical analysis (N = 1)). (DOCX) [file pone.0242168.s003.docx]

**Supporting Table S3. Typing resolution and number of reads.** Typing status according to number of reads (mean [min-max]) (No.: number) and read depth (mean [min-max]); (Incorrectly typed samples could not be included in the statistical analysis (N=1)).

| Typing status | No. of Reads | Read depth | p-value |
| --- | --- | --- | --- |
| DO Typed | 950 [662-1304] | 12 [8-17] | <0.001 |
| DO Ambiguous | 746 [381-974] | 9 [5-12] |  |
| DO Unresolved | 757 [693-821] | 10 [9-10] |  |
| DO Incorrect | 732 | 9 |  |
| YT Typed | 704 [477-1563] | 10 [7-22] | <0.001 |
| YT Unresolved | 493 [316-623] | 7 [5-9] |  |
| DI Typed | 1469 [901-3220] | 12 [7-26] | <0.001 |
| DI Ambiguous | 1104 [678-1410] | 9 [6-12] |  |
| FY Typed | 584 [396-1176] | 10 [7-21] | <0.001 |
| FY Ambiguous | 442 [433-451] | 8 [8-8] |  |
| FY Unresolved | 448 [224-590] | 8 [4-10] |  |
| FY Incorrect | 442 | 8 |  |
| JK Typed | 2896 [1974-5025] | 11 [8-20] | <0.001 |
| JK Ambiguous | 2388 [1806-2984] | 9 [7-12] |  |
| JK Unresolved | 2005 [1028-2306] | 8 [4-9] |  |
| JK Incorrect | 2250 | 9 |  |
| LW Typed | 483 [200-1243] | 11 [5-29] | - |
| LW Ambiguous | 372 [323-421] | 9 [7-10] |  |
| CO Typed | 1315 [815-2690] | 11 [7-23] | 0.005 |
| CO Ambiguous | 1161 [951-1515] | 10 [8-13] |  |
| CO Unresolved | 880 [648-1055] | 8 [6-9] |  |
| K Typed | 1742 [1132-2721] | 14 [9-21] | <0.001 |
| K Ambiguous | 1326 [1013-1927] | 10 [8-15] |  |
| K Unresolved | 1302 [1087-1697] | 10 [9-13] |  |
| K Incorrect | 1938 | 15 |  |
| IN Typed | 5659 [4175-9360] | 13 [9-21] | <0.001 |
| IN Ambiguous | 4567 [2337-6245] | 10 [5-14] |  |
| IN Unresolved | 5279 [4709-6412] | 12 [10-14] |  |
