# Supplementary material for: Blood group typing from whole-genome sequencing data
Source: PLoS One. 2020 Nov 12;15(11):e0242168. doi: 10.1371/journal.pone.0242168 (PMC7660531; doi:10.1371/journal.pone.0242168)
Supplement: S4 Table — WGS blood group results ambiguities (No.: Number). (DOCX) [file pone.0242168.s004.docx]

**Supporting Table S4. Typing ambiguities.** WGS blood group results ambiguities (No.: number)

| KEL*02 ambiguities | No. |
| --- | --- |
| KEL*02.01 KEL*02.01M KEL*02.09N | 1 |
| KEL*02.01 KEL*02.02M KEL*02.05N KEL*02.10M KEL*02.19N KEL*02.21N KEL*02.25N | 1 |
| KEL*02.01 KEL*02.02M KEL*02.05N KEL*02.14N KEL*02.19N | 1 |
| KEL*02.01 KEL*02.02M KEL*02.05N KEL*02.14N KEL*02.19N KEL*02.21N KEL*02.22 KEL*02.25N | 1 |
| KEL*02.01 KEL*02.02M KEL*02.05N KEL*02.19N KEL*02.25N | 1 |
| KEL*02.01 KEL*02.02N KEL*02.04M KEL*02.08M KEL*02.14N KEL*02.18.01 KEL*02.18.02 | 1 |
| KEL*02.01 KEL*02.02N KEL*02.05M KEL*02.07M KEL*02.09M KEL*02.18.01 KEL*02.18.02 | 1 |
| KEL*02.01 KEL*02.02N KEL*02.09N KEL*02.18.01 KEL*02.18.02 KEL*02.36 | 1 |
| KEL*02.01 KEL*02.02N KEL*02.18.01 KEL*02.18.02 | 1 |
| KEL*02.01 KEL*02.03 KEL*02.05N KEL*02.10M KEL*02.13 KEL*02.14N KEL*02.17N KEL*02.19N KEL*02.21 KEL*02.22 KEL*02.23 KEL*02.24N | 1 |
| KEL*02.01 KEL*02.03 KEL*02.10M KEL*02.11N KEL*02.12N KEL*02.13N KEL*02.14N KEL*02.17 KEL*02.21 KEL*02.22 KEL*02.23 KEL*02.30 KEL*02.31 KEL*02.37 | 1 |
| KEL*02.01 KEL*02.03 KEL*02.21 | 1 |
| KEL*02.01 KEL*02.04M KEL*02.17N KEL*02.18.01 KEL*02.18.02 KEL*02.21N KEL*02.25N KEL*02.33 | 1 |
| KEL*02.01 KEL*02.05M KEL*02.07M KEL*02.09M KEL*02.11N KEL*02.12N KEL*02.13 KEL*02.13N KEL*02.14N KEL*02.17 KEL*02.22 KEL*02.30 KEL*02.31 KEL*02.37 | 1 |
| KEL*02.01 KEL*02.07M KEL*02.09M KEL*02.29 | 1 |
| KEL*02.01 KEL*02.08M KEL*02.10 KEL*02.10N KEL*02.16N KEL*02.19 | 1 |
| KEL*02.01 KEL*02.08M KEL*02.10 KEL*02.11M KEL*02.15N KEL*02.16N KEL*02.19 KEL*02.24N KEL*02.26 KEL*02.27N KEL*02.32 KEL*02.33 | 1 |
| KEL*02.01 KEL*02.08N KEL*02.09M KEL*02.14.01 KEL*02.14.02 KEL*02.14N KEL*02.22 KEL*02.24 | 1 |
| KEL*02.01 KEL*02.08N KEL*02.14.01 | 1 |
| KEL*02.01 KEL*02.08N KEL*02.14.01 KEL*02.14.02 KEL*02.24 | 1 |
| KEL*02.01 KEL*02.09M KEL*02.10M KEL*02.22N KEL*02.25 KEL*02.25N KEL*02.27 KEL*02.28 KEL*02.34 KEL*02.36 | 1 |
| KEL*02.01 KEL*02.10M KEL*02.20N | 1 |
| KEL*02.01 KEL*02.10N KEL*02.14N KEL*02.22 KEL*02.23 | 1 |
| KEL*02.01 KEL*02.11M KEL*02.13 KEL*02.14N KEL*02.15N KEL*02.22 KEL*02.26 KEL*02.32 KEL*02.33 | 1 |
| KEL*02.01 KEL*02.12 KEL*02.12N KEL*02.13N KEL*02.14N KEL*02.22 KEL*02.25N | 1 |
| KEL*02.01 KEL*02.12 KEL*02.20N KEL*02.23 KEL*02.24N KEL*02.27N | 1 |
| KEL*02.01 KEL*02.12N KEL*02.13N KEL*02.15N KEL*02.26 KEL*02.30 KEL*02.32 | 1 |
| KEL*02.01 KEL*02.13 KEL*02.14N KEL*02.22 | 2 |
| KEL*02.01 KEL*02.13 KEL*02.14N KEL*02.22 KEL*02.24N | 1 |
| KEL*02.01 KEL*02.14.01 KEL*02.14.02 KEL*02.22N KEL*02.24 KEL*02.25 KEL*02.27 KEL*02.28 KEL*02.34 | 1 |
| KEL*02.01 KEL*02.14.01 KEL*02.14.02 KEL*02.24 | 1 |
| KEL*02.01 KEL*02.14N | 1 |
| KEL*02.01 KEL*02.14N KEL*02.22 | 2 |
| KEL*02.01 KEL*02.14N KEL*02.22 KEL*02.23 | 1 |
| KEL*02.01 KEL*02.19 | 1 |
| KEL*02.01 KEL*02.21N KEL*02.25N | 1 |
| KEL*02.01 KEL*02.27N | 1 |
| KEL*02.01 KEL*02.30 | 1 |
| KEL*02.01 KEL*02.33 | 1 |
| KEL*02.01 KEL*02.36 | 2 |
| KEL*02.01 KEL*02.36 | 1 |
| Total | 44 |
| FY*01 ambiguities | N |
| FY*01.01 FY*01.03W FY*01.07N | 1 |
| Total | 1 |
| FY*02 ambiguities | N |
| FY*02.01 FY*02.01.02W | 1 |
| Total | 1 |
| JK*01 ambiguities | N |
| JK*01.01 JK*01.02N JK*01.04W | 1 |
| JK*01.01 JK*01.05W JK*01.07N | 1 |
| JK*01.01 JK*01.01W | 1 |
| JK*01.01 JK*01.01W JK*01.10N | 1 |
| JK*01.01 JK*01.03N | 1 |
| JK*01.01 JK*01.04N | 1 |
| JK*01.01 JK*01.07N | 1 |
| JK*01.01 JK*01.08N | 1 |
| JK*01.01 JK*01.10N | 1 |
| JK*01.01 JK*01.02W JK*01.03N JK*01.05N JK*01.07N | 1 |
| Total | 10 |
| JK*02 ambiguities | N |
| JK*02.01 JK*02.01N JK*02.02N | 2 |
| JK*02.01 JK*02.01N JK*02.02N JK*02.04N JK*02.09N JK*02.10N | 1 |
| JK*02.01 JK*02.01W JK*02.11N JK*02.13N | 1 |
| JK*02.01 JK*02.02W JK*02.05N | 1 |
| JK*02.01 JK*02.02W JK*02.06N JK*02.07N | 1 |
| JK*02.01 JK*02.04N | 1 |
| JK*02.01 JK*02.04N JK*02.09N JK*02.10N | 1 |
| JK*02.01 JK*02.06N | 1 |
| JK*02.01 JK*02.08N | 3 |
| Total | 12 |
| DO*01 ambiguities | N |
| DO*01.01 DO*01.01N | 1 |
| DO*01.01 DO*01.01N DO*01.08 | 1 |
| DO*01.01 DO*01.02N DO*01.05 DO*01.06 DO*01.08 | 1 |
| DO*01.01 DO*01.02N DO*01.05 DO*01.10 | 1 |
| DO*01.01 DO*01.08 | 1 |
| DO*01.01 DO*01.08 DO*01.09 | 1 |
| DO*01.01 DO*01.10 | 1 |
| Total | 7 |
| DO*02 ambiguities | N |
| DO*02.01 DO*02.01N DO*02.02N | 1 |
| DO*02.01 DO*02.01N DO*02.03N | 1 |
| DO*02.01 DO*02.02N | 1 |
| DO*02.01 DO*02.03N DO*02.04 | 1 |
| DO*02.01 DO*02.04 DO*02.04N | 1 |
| DO*02.01 DO*02.04 DO*02.04N DO*02.07 DO*02.08 | 1 |
| DO*02.01 DO*02.07 | 1 |
| DO*02.01 DO*02.07 DO*02.08 | 1 |
| DO*02.01 DO*02.08 | 1 |
| Total | 9 |
| IN*02 ambiguities | N |
| IN*02.01 IN*02.03 | 10 |
| IN*02.01 IN*02.03 IN*02.04 | 2 |
| IN*02.01 IN*02.03 IN*02.05 IN*02.06 | 1 |
| IN*02.01 IN*02.03 IN*02.06 | 4 |
| IN*02.01 IN*02.04 | 1 |
| IN*02.01 IN*02.04 IN*02.05 | 2 |
| IN*02.01 IN*02.04 IN*02.06 | 1 |
| IN*02.01 IN*02.05 | 2 |
| IN*02.01 IN*02.06 | 1 |
| Total | 24 |
| CO*01 ambiguities | N |
| CO*01.01 CO*01.02N | 5 |
| CO*01.01 CO*01.03N | 3 |
| CO*01.01 CO*01.03N CO*01.06N | 2 |
| CO*01.01 CO*01.04 | 1 |
| CO*01.01 CO*01.04N | 1 |
| Total | 12 |
| DI*02 ambiguities | N |
| DI*02.01 DI*02.01N | 1 |
| DI*02.01 DI*02.01N DI*02.03 DI*02.08 DI*02.11 DI*02.12 DI*02.14.01 DI*02.20 DI*02.21 | 1 |
| DI*02.01 DI*02.01N DI*02.08 | 1 |
| DI*02.01 DI*02.01N DI*02.09 DI*02.10 DI*02.15 DI*02.17 DI*02.18 DI*02.20 | 1 |
| DI*02.01 DI*02.03 DI*02.05 DI*02.06 DI*02.07 DI*02.11 DI*02.12 DI*02.13 DI*02.14.01 DI*02.15 DI*02.19 DI*02.21 | 1 |
| DI*02.01 DI*02.03 DI*02.08 DI*02.11 DI*02.12 | 1 |
| DI*02.01 DI*02.03 DI*02.11 DI*02.12 | 1 |
| DI*02.01 DI*02.03 DI*02.11 DI*02.12 DI*02.14.01 DI*02.21 | 1 |
| DI*02.01 DI*02.05 DI*02.07 DI*02.09 DI*02.13 DI*02.15 DI*02.17 DI*02.18 | 1 |
| DI*02.01 DI*02.06 | 2 |
| DI*02.01 DI*02.06 DI*02.19 | 1 |
| DI*02.01 DI*02.06 DI*02.07 DI*02.10 DI*02.17 DI*02.18 DI*02.19 | 1 |
| DI*02.01 DI*02.06 DI*02.07 DI*02.13 DI*02.19 | 2 |
| DI*02.01 DI*02.06 DI*02.07 DI*02.19 | 1 |
| DI*02.01 DI*02.06 DI*02.09 DI*02.10 DI*02.15 DI*02.17 DI*02.18 | 1 |
| DI*02.01 DI*02.08 | 2 |
| DI*02.01 DI*02.08 DI*02.10 DI*02.14.01 DI*02.21 | 1 |
| Total | 20 |
| LW*05 ambiguities | N |
| LW*05.01 LW*05.01N | 2 |
| Total | 2 |
