# Supplementary material for: Blood group typing from whole-genome sequencing data
Source: PLoS One. 2020 Nov 12;15(11):e0242168. doi: 10.1371/journal.pone.0242168 (PMC7660531; doi:10.1371/journal.pone.0242168)
Supplement: S5 Table — Number of polymorphisms revealed by whole-genome analysis but not described in the ISBT database (observed in at least 5 samples with a minimum coverage of 10 reads). (DOCX) [file pone.0242168.s005.docx]

**Supporting Table S5. Unreported polymorphisms.** Number of polymorphisms revealed by whole-genome analysis but not described in the ISBT database (observed in at least 5 samples with a minimum coverage of 10 reads)

| Gene | Intron | Exon | Total |
| --- | --- | --- | --- |
| *AQP1* (CO) | 25 | 0 | 25 |
| *SLC4A1* (DI) | 18 | 0 | 18 |
| *ART4* (DO) | 10 | 2 | 12 |
| *ACKR1* (FY) | 10 | 0 | 10 |
| *CD44* (IN) | 98 | 2 | 100 |
| *SLC14A1* (JK) | 80 | 1 | 81 |
| *KEL* | 2 | 0 | 2 |
| *ICAM4* (LW) | 10 | 0 | 10 |
| *ACHE* (YT) | 9 | 0 | 9 |
| Total | 262 | 5 | 267 |
