# Supplementary material for: Blood group typing from whole-genome sequencing data
Source: PLoS One. 2020 Nov 12;15(11):e0242168. doi: 10.1371/journal.pone.0242168 (PMC7660531; doi:10.1371/journal.pone.0242168)
Supplement: S6 Table — Description of exonic SNPs revealed by whole-genome analysis. Note that mutations in IN are located after the codon stop (exon 9) in IN isoform 4 described in ISTB. (DOCX) [file pone.0242168.s006.docx]

**Supporting Table S6. New polymorphisms in exons.** Description of exonic SNPs revealed by whole-genome analysis. Note that mutations in IN are located after the codon stop (exon 9) in IN isoform 4 described in ISTB.

| Gene and  position | Exon | AA change | Accession Number | No. |
| --- | --- | --- | --- | --- |
| *ART4* (DO) |  | - |  |  |
| T378C | 2 | - | LS997580.1 | 18 |
| C624T | 2 | - | LS997580.1 | 21 |
| *CD44* (IN) |  |  |  |  |
| A1250G | 10 | p.Lys417Arg | AB468969.1 | 37 |
| T1436C | 12 | p.Ile479Tyr | AH003670.2 | 41 |
| *SLC14A1* (JK) |  | - |  |  |
| A588C | 4 |  | JN410950.1 | 37 |
