# Supplementary material for: Blood group typing from whole-genome sequencing data
Source: PLoS One. 2020 Nov 12;15(11):e0242168. doi: 10.1371/journal.pone.0242168 (PMC7660531; doi:10.1371/journal.pone.0242168)
Supplement: Attachment — Submitted filename: PONE-D-20-17434.docx [file pone.0242168.s007.docx]

**Manuscript number PONE-D-20-17434**

The study aimed to validate red blood cell antigen typing from WGS data using a software validated for HLA typing.

Seventy – nine (79) samples, representing male Afghan volunteers, were used for the study. The samples had been tested by SNaPshot analysis. This SNaPshot analysis genotyped for SNVs associated with major blood group antigen polymorphisms for the nine blood groups analysed in this study.

The WGS data was compared to the previous published SNaPshot genotyping data.

WGS also revealed other variants including the presence of JK allelic variants associated with weak Jk antigen expression as well five SNVs in exonic regions for DO, IN and JK variants that have been described in Genebank but for which the blood group phenotype association is unknown. The limitation of the study is that no serology was performed and the authors note this in the discussion.

The study also revealed a number of intronic variants which could be informative in future population studies.

**Major comments**

1	The NGS data correlated with SNaPshot in defining alleles that were homozygous for the target SNV however there were five (102/107) incorrect typing results at the heterozygous positions. Of note, also a large number of positions (both homozygous and heterozygous) could not be called.

The authors should discuss whether the incorrect typing at heterozygous positions as well as this level of calls “not defined” is acceptable. The mean read depth of the genome was 11.8 which is not high and a reflection of the WGS approach used. Is this arguing for a need for a more targeted and efficient approach to obtain a higher read depth?

2	The approach of using the HLA software to interpret the blood group (Other than ABO) data appears novel although the study appears more a feasibility study than a validation study at this stage.

3	Please check the bi-allelic polymorphisms listed in the Material and Methods under Blood group genotyping e.g KEL, JK , etc. as a few are now not matching the most recently revised ISBT reference tables.

**Other comments:**

Results Page 11 Line 210 should the number be ‘148’ not ‘146’?

Please also check the ISBT terminology used e.g. use Jk (a+^w^ ) phenotype

**Discussion**

First paragraph, last sentence: Please consider including an explanation for not including MNS because this was included in the study SNaPshot study.

The authors state in the discussion that the “results showed 99.5% of concordance for blood group polymorphisms” – please show how they arrived at this figure.

**Editorial**

Page 11 Line 189 ‘WGS-based typing targeting all…’ Space between targeting and all

Same paragraph – last sentence – do the authors mean ‘confirm or refute’…?
