# Supplementary material for: Blood group typing from whole-genome sequencing data
Source: PLoS One. 2020 Nov 12;15(11):e0242168. doi: 10.1371/journal.pone.0242168 (PMC7660531; doi:10.1371/journal.pone.0242168)
Supplement: Attachment — Submitted filename: reply to rebuttal letter PONE D2017434_092120.docx [file pone.0242168.s008.docx]

Dr. Di Cristofaro

UMR7268

Faculté de Médecine Timone

Marseille France

Santosh K. Patnaik, MD, PhD

Academic Editor

PLOS ONE

PONE-D-20-17434

BLOOD GROUP TYPING FROM WHOLE-GENOME SEQUENCING DATA

PLOS ONE

To the Editor and the Reviewers,

Thank you for having considered our manuscript for publication to PLOS ONE. We are grateful to both reviewers for their careful review. Accordingly, we are submitting a revised version of our manuscript that addresses the points raised during the review process.

Please, find in this letter responses to each point raised by the editor and the reviewers.

Editor comments:

**1. Please ensure that your manuscript meets PLOS ONE's style requirements, including those for file naming.**

*> File naming and authors’ affiliations have been modified according to PLOS ONE style requirements.*

**2. Please provide additional details regarding participant consent. In the ethics statement in the Methods and online submission information, please clarify what type of consent you obtained (for instance, written or verbal, and if verbal, how it was documented and witnessed).**

*> Type of consent obtained from participants was clarified in the ethics statement in the Methods and online submission information as follows: « All samples were obtained from unrelated male Afghan volunteers after obtaining written informed consent. The study protocol was registered by the Ministere de l’Enseignement Superieur et de la Recherche in France (committee 208C06, decision AC-2008-232). Institutional review board Ministere de l’Enseignement Superieur et de la Recherche in France committee 208C06, (decision AC-2008-232) specifically approved this study. »*

**3. To comply with PLOS ONE submission guidelines, in your Methods section, please provide additional information regarding your statistical analyses.**

*> Additional information regarding statistical analyses was added in the Methods section as follows : « Statistical analyses were performed with GRAPH PAD Prism 5 software (California USA,* [*www.graphpad.com*](http://www.graphpad.com/)*). Number of reads are presented as mean and range [min, max]. Differences among number of reads according to typing gene status were tested using Kruskal-Wallis one-way ANOVA for three values and Mann Whitney test for two values. Threshold for significance (alpha) was set at 0.05.  »*

**4. Financial Disclosure section, Funding Statement and Competing Interests Statement modifications**

*>Funding, Financial disclosure, disclosure statement and authors’ contributions sections have been added to the manuscript as follows:*

*FUNDING*

*No funding was received for this research. The funder provided support in the form of salaries for authors JP, PG and PN, but did not have any additional role in the study design, data collection and analysis, decision to publish, or preparation of the manuscript. The specific roles of these authors are detailed in the ‘author contributions’ section.*

*Financial Disclosure*

*The authors received no specific funding for this work. Authors Julien Paganini and Philippe Gouret are employed by a commercial company: Xegen, Gemenos, France. Author Peter L. Nagy is employed by a commercial company: Praxis Genomics LLC, Atlanta, Georgia, USA.*

*Disclosure Statement*

*The authors have no conflicts of interest to declare. Commercial affiliation of JP, PG and PN does not alter our adherence to all PLOS ONE policies on sharing data and materials.*

*Author Contributions*

*Conceptualization, Methodology, Visualization and Writing – Original Draft Preparation : JP and JDC; Data Curation and Formal Analysis : JP, PN, NR, PG and JDC ; Investigation Resources : CP, JC and JDC ; Project Administration : CP and JC ; Funding Acquisition : PN and JC ; Software : JP and PG ; Supervision and Validation: JC and CP ; Writing – Review & Editing : all authors*

**Reviewers’ comments:**

**The authors would like to thank both reviewers for their accurate review and kindness, their knowledge of blood groups have improved our manuscript.**

**Reviewer #1**

**Actual source NGS sequences do not appear to be publicly available.**

*> Authors agree with the reviewer. The reference of NGS has been added in the introduction and material section (Determination of the phylogenetic origins of the Árpád Dynasty based on Y chromosome sequencing of Béla the Third. Nagy PL, et al. Eur J Hum Genet. 2020 Jul 7. PMID: 32636469). The link for uploaded data has been added in the results section: “Sequencing data are available at* [*http://www.ncbi.nlm.nih.gov/bioproject/662371*](http://www.ncbi.nlm.nih.gov/bioproject/662371)*”. This link will become public once the paper is published. A reviewer link is also normally created once the data set is fully processed as well.*

**Although the authors provide a thorough description of the bioinformatic pre-alignment process, there is no mention of the aligner used, the specific aligning parameters, and the human genome build employed in this study. Is this because the alignment is done by the PolyPheMe software as well?**

*> Authors agree with the reviewer. Additional information regarding alignment process was added in the Material and Methods section as follows: “WGS data were directly aligned to each gene as reference, no human genome was used for read mapping. Alignments were generated in PolyPheMe software with a Bowtie tool (*[*Langmead*](https://www.ncbi.nlm.nih.gov/pubmed/21154709) *et al. 2009; Langmead 2010).”*

**The manuscript reports a potential novel weak FY allele, which on Table S1 is described as c.125A (p.Gly42Asp – the Fyb antigen) with c.298G>A (p.Ala100Thr) in cis. The presence of the Ala100Thr variant alone in a Fy(b) background was in fact reported previously in the literature (Olsson et al, BJH 1998, 103, 1184-1191 9886340) with no reported weakening of FY expression.**

*> Authors thank the reviewer. Mention of weak FY phenotype was deleted concerning this FY allele with c.125A and c.298G>A in cis. Olsson et al. has been added as reference.*

*Results and Tables were modified accordingly: “FY*02 allele associated with c.298G>A (p.Ala100Thr) was found in 18 samples (Olsson et al.; 1998).”*

*Discussion was modified as follows: “ We were able to type the JK*01W.01 allele (Jk (a+w))* *(**19) and the FY*02 allele associated with c.298G>A (p.Ala100Thr)* *(Olsson et al.; 1998)**.”*

**Four novel missense variants are reported by the study. For the Dombrock blood group the authors report p.Asp265Asn as a novel change; however, this actually corresponds to the known Do(a/b) antithetical antigens. This is however, one of the instances where the human genome build reference nucleotide does not match the ISBT reference table. In hg38, the reference nucleotide in chr12: 14840505 is a C, which leads to Asp265 (ART4 is coded in the minus strand). However, for immunohematologists and in the ISBT database, Asn265 is considered the reference.**

**The manuscript also lists p.Pro179Arg as a novel missense change in the JK blood group, however this variant is listed as part of JK*02N.13 in the most recent ISBT JK database (v6.0 01-MAR-2020 v2.0). Was this variant identified in cis with p.Asp280Asn (Jkb)? Was it identified without concomitant p.Met167Val, which is part of the definition of JK*02N.13?**

*> Authors thank the reviewer. We made a mistake when we used genebank sequences for translation. The coordinate was checked and corrected. DO and JK new polymorphisms induce no amino-acid change. Authors apologize for these mistakes. Mutations in IN gene were double checked. They are located after the codon stop (exon 9) in IN isoform 4 described in ISTB. This information has been added to the Supporting Table S6 legend.*

*Text and tables have been modified accordingly throughout the manuscript.*

**The manuscript clearly reports the number of cases were heterozygosity was missed, and mentions that this was associated with low depth. It would be useful to provide the range of depth that was associated with this phenomenon, to aid in the determination of a minimum depth for this particular approach.**

*> Authors thank the reviewer. Range of depth associated with incorrectly typed heterozygous samples was added in Supporting Table S3. Discussion has been modified accordingly: ”WGS data quality is assessed by the estimation of read depth. A former study conducted on WGS data established a minimum of 15x for RBC antigen typing in the clinical field* *(**6, 14)**. Here, mean read depth* *of the genome was estimated at 11.8**x [5.5x-18.4x]* *and* *read depth* *for each gene reached higher values.* *For each gene, typing resolution was significantly associated with the number of reads mapped on its sequence and* *ambiguous and incorrectly typing showed low numbers of reads corresponding to the missing allele and read depth equal to or below 15x. Our study thus confirms that RBC typing from WGS should be considered reliable with read depths strictly above 15x. To reach this goal, genome sequencing of one human (3Gb) should be analyzed with at least 45 Gb of data, here mean data was 34 Gb [16-53]”. Also, this sentence has been added in the abstract: “Our study confirms that RBC typing from WGS should be considered reliable with read depths strictly above 15x.”*

**Minor revisions:**

**- Page 11, line, ‘targetingall’ is missing a space.**

**- Page 15, second paragraph, first line – the subject is singular (‘laboratory’) while the verb is plural (‘are’).**

*> Authors thank the reviewer, typos have been corrected.*

**Reviewer #2**

**Major comments**

**1 The authors should discuss whether the incorrect typing at heterozygous positions as well as this level of calls “not defined” is acceptable. The mean read depth of the genome was 11.8 which is not high and a reflection of the WGS approach used. Is this arguing for a need for a more targeted and efficient approach to obtain a higher read depth?**

*> Authors agree with the reviewer, the corresponding paragraph has been modified as follows: ” WGS data quality is assessed by the estimation of read depth. A former study conducted on WGS data established a minimum of 15x for RBC antigen typing in the clinical field (6, 14)**. Here, mean read depth* *of the genome was estimated at 11.8**x [5.5x-18.4x]* *and* *read depth* *for each gene reached higher values.* *For each gene, typing resolution was significantly associated with the number of reads mapped on its sequence and* *ambiguous and incorrectly typing showed low numbers of reads corresponding to the missing allele and read depth equal to or below 15x. Our study thus confirms that RBC typing from WGS should be considered reliable with read depths strictly above 15x. To reach this goal, genome sequencing of one human (3Gb) should be analyzed with at least 45 Gb of data, here mean data was 34 Gb [16-53].”*

*This sentence has been added in the abstract: “Our study confirms that RBC typing from WGS should be considered reliable with read depths strictly above 15x.”*

**2 The approach of using the HLA software to interpret the blood group (Other than ABO) data appears novel although the study appears more a feasibility study than a validation study at this stage.**

*> Authors agree with the reviewer, abstract and introduction have been modified accordingly.*

**3 Please check the bi-allelic polymorphisms listed in the Material and Methods under Blood group genotyping e.g KEL, JK , etc. as a few are now not matching the most recently revised ISBT reference tables.**

*> Authors agree with the reviewer, ISBT Terminology has been checked.*

**Other comments:**

**Results Page 11 Line 210 should the number be ‘148’ not ‘146’?**

**Please also check the ISBT terminology used e.g. use Jk (a+w ) phenotype.**

*> Authors agree with the reviewer, the number has been corrected, also abstract, results and discussion sections have been modified accordingly: “Our results showed 93% of concordance for blood group polymorphisms and 91% for HLA-DRB1.”*

*ISBT Terminology has been checked.*

**Discussion**

**First paragraph, last sentence: Please consider including an explanation for not including MNS because this was included in the study SNaPshot study.**

*> Authors agree with the reviewer. As for RHD/RHCE system, MNS could not be included because of the existence of hybrids. The sentence has been modified accordingly: “Whereas targeted strategies, such as PCR followed by sequencing or SnaPshot, circumvent specificity issues of genes with structural changes and hybrids such as RHCE/RHD and GPA/GPB ; their analysis from WGS data requires specific bioinformatic approaches including CNV (copy number variation) analysis. Therefore, such systems were not included in this study.”*

**The authors state in the discussion that the “results showed 99.5% of concordance for blood group polymorphisms” – please show how they arrived at this figure.**

*> WGS analyses allow identification of 972 SNPs; 967 SNP were correctly identified (i.e. 99.5%). 93.4% of concordance was obtained when taking into account ambiguous typing. The sentence in the discussion was modified as follows: “Our results showed that blood group typing deduced from WGS were correct at 99.5% compared to SNaPshot analysis (967 SNP correctly identified out of 972 typed); 93% of concordance was obtained when taking into account ambiguous typing. In a clinical or research context however, ambiguous RBC results need to be reanalyzed. HLA-DRB1 typing from WGS showed 91% of concordance with those obtained by amplicon-based monoallelic sequencing.”*

**Editorial**

**Page 11 Line 189 ‘WGS-based typing targeting all…’ Space between targeting and all**

**Same paragraph – last sentence – do the authors mean ‘confirm or refute’…?**

*> Authors thank the reviewer, modifications have been made.*

The authors would like to thank reviewers for their critical review, we feel that our manuscript has been improved. We look forward to the editor and reviewers’ comments and the editorial board’s decision.

Please do not hesitate to contact us if you require any further information.

Yours sincerely,
