# Supplementary material for: Blood group typing from whole-genome sequencing data
Source: PLoS One. 2020 Nov 12;15(11):e0242168. doi: 10.1371/journal.pone.0242168 (PMC7660531; doi:10.1371/journal.pone.0242168)
Supplement: Attachment — Submitted filename: reply to rebuttal letter PONE D2017434_R2_102620.docx [file pone.0242168.s009.docx]

Dr. Di Cristofaro

UMR7268

Faculté de Médecine Timone

Marseille France

Santosh K. Patnaik, MD, PhD

Academic Editor

PLOS ONE

PONE-D-20-17434R1

BLOOD GROUP TYPING FROM WHOLE-GENOME SEQUENCING DATA

PLOS ONE

To the Editor and the Reviewer,

Thank you for having considered our revised manuscript for publication to PLOS ONE. We are grateful to reviewer for her/his second review. Accordingly, we are submitting a revised version of our manuscript.

Please, find in this letter responses to each point raised by the reviewer.

*******

**Reviewer #2**

**The authors have clarified some of the queries with regard to Incorrect Typing and with regard to Blood Group Nomenclature however anomalies still remain in both regards.**

**Abstract the Conclusion:Page 2 Line 31 – 32, The authors should reconsider the conclusion which is too general for the results presented in this study. The stated aim includes estimating the feasibility of RBC antigen typing from WGS data.**

**Table 2 shows the authors find incorrect typing results for 5 samples and correct results for 102 samples with heterozygous calls. They also find a large number of not determined (unresolved) results with 53 homozygous samples and 10 heterozygous not resolved.**

**The authors elsewhere show that the read depth is insufficient at 11.8 which supports previous findings that read depth needs to be above 15.**

**The conclusion is that RBC antigen typing is feasible however improvements in read depth are needed to improve the accuracy in typing for SNV polymorphisms.**

**The paper does show the potential for WGS in detecting other alleles, such as the weak JK alleles, with potential to be used to screen for rare donors.**

*> Authors agree with the reviewer, the abstract has been modified: “Incorrect typing and unresolved results confirm that WGS should be considered reliable with read depths strictly above 15x. Our results supported that RBC antigen typing from WGS is feasible but requires improvements in read depth for SNV polymorphisms typing accuracy. We also showed the potential for WGS in screening donors with rare blood antigens, such as weak JK alleles. The development of WGS analysis in immunogenetics laboratories would offer personalized care in the management of RBC disorders.*

**Nomenclature:**

**Materials and Methods:**

**Page 5 line 84 & 85 the bracketed list gives the internationally HUGO blood group gene names with the Blood Group System symbols in brackets. However the gene names given for FY, IN, CO DI and LW are incorrect. The gene names for these Blood Group Systems are ACKR1 (FY), CD44 (IN), AQP1 (CO), SLC4A1 (DI) and ICAM4 (LW).**

**Reference** [**http://www.isbtweb.org/fileadmin/user_upload/Table_of_blood_group_systems_v6.0_6th_August_2019.pdf**](http://www.isbtweb.org/fileadmin/user_upload/Table_of_blood_group_systems_v6.0_6th_August_2019.pdf)

*> Authors thank the reviewer, gene names have been corrected.*

**Page 5 Line 87 to 89: After “Fourteen SNPs were analysed corresponding to bi-allelic polymorphisms” the authors show the following amino acid changes (phenotypes). The Duffy needs editing to match ISBT Tables as follows: Page 5 Line 89: The phenotype for (FY p.Arg89Cys) is Fya+w, and the phenotype for the FY promoter change (p.0) with the FYc-67T>C change is (Fy(a-b-) erythroid cells only)**

*> Authors thank the reviewer, phenotypes have been corrected.*

**Presentation of data in Table 2:**

**There are ambiguities in the presentation of data in Table 2. For example for FY the third column and fourth column indicate that the FY*01N.01 allele showed Homozygous Correct Typing for 64 cases with 8 not defined. This is generally a rare allele. The next line shows for the FY*02 allele (the alternate for the FY*01) that 32 were Homozygous. Yet there are only 79 individuals in the study and these numbers as written exceed the number in the study (64+ 32). This is not counting the 26 Heterozygous cases and 7 incorrect or undefined. The next line indicates for FY*0W.01 there are a further 61 cases homozygous (now there are 64 + 32 + 61). Again in Table 2, The authors also report 70 Homozygous for IN*01 which is indeed a very rare allele and should, if confirmed, be commented on.**

*> Authors thank the reviewer, ambiguities in table 2 was due to allele names instead of polymorphisms. No sample was typed FY*01N.01; among the 72 homozygous samples (wild type as shown in Table 1) at position ACKR1 (-67T>C), 64 samples were correctly defined and 8 samples were not defined. Among the 35 samples homozygous at position ACKR1 (125G>A), whether wild type or mutated, 32 were correctly defined and 3 were not defined. Among the 30 samples heterozygous at the same position, 26 samples were correctly typed, 1 sample was not correctly typed and 3 samples were not defined. No sample was typed IN*01. Among the 72 homozygous samples (wild type as shown in Table 1) at position CD44* (137G>C)*, 70 samples were correctly defined and 2 were not defined.*

*Allele names have been replaced with polymorphisms in Table 2.*

**References**

**Introduction Line 46: Suggest include the papers by Lane et al with (1,2) as well as in the next paragraph.**

*> Authors agree with the reviewer, reference has been added*

**Minor Edits:**

**Introduction: Line 3: Research and CE-environment labs – suggest Research and Clinical laboratories working within regulatory approved frameworks e.g. Council of Europe (CE).**

*> Authors agree with the reviewer, text has been modified accordingly.*

The authors would like to thank the reviewer. We look forward to the editor and reviewer’s comments and the editorial board’s decision.

Please do not hesitate to contact us if you require any further information.

Yours sincerely,
